# Dose Titration of Plant‐Based Flavonoid Blend Supplementation on Performance, Digestibility, Gut Microbiome, Blood Biomarkers, and Meat Quality of Growing Rabbits

**DOI:** 10.1002/fsn3.72156

**Published:** 2026-07-28

**Authors:** Md. Aliar Rahman, Md. Mehedi Hasan, Md. Abul Hashem, Mahbubul Pratik Siddique, Rakhi Chowdhury

**Affiliations:** ^1^ Department of Animal Nutrition, Faculty of Animal Husbandry Bangladesh Agricultural University Mymensingh Bangladesh; ^2^ Department of Animal Science, Faculty of Animal Husbandry Bangladesh Agricultural University Mymensingh Bangladesh; ^3^ Department of Microbiology and Hygiene, Faculty of Veterinary Science Bangladesh Agricultural University Mymensingh Bangladesh

**Keywords:** growth, liver health, meat quality, microbiota, nutrients utilization, phytobiotics, rabbits

## Abstract

Rabbit meat is increasingly valued for its high nutritional quality, driving growing interest in enhancing its production through phytobiotics like plant‐based flavonoid blend (PFB). So, this study looked at how a PFB at different doses influences the performance, digestibility, gut microbiota, blood biomarkers, liver health, and meat quality of growing rabbits. Sixty rabbits (body weight 552.63 ± 13.44 g) were assigned at random to five dietary groups, having twelve replicates per group. The rabbits were fed a basal diet as a total mixed ration having 17.02% crude protein and 11.60 MJ metabolizable energy/kg dry matter (DM), supplemented with PFB (g/kg diet) at 0.0 (control), 0.20, 0.40, 0.60, and 0.80. Following a 2 weeks adjustment period, the feeding trial was conducted for 5 weeks. When the trial was over, rabbits were sacrificed to collect digesta, blood, and meat samples for further analysis. Supplementation with PFB at 0.60 g/kg diet (range: 0.50–0.70 g/kg) showed a better final body weight (*p* = 0.01), weight gain (*p* < 0.001), and feed conversion ratio (*p* < 0.001), while significantly improving DM, nitrogen‐free extract (*p* < 0.001), and ether extract (*p* = 0.02) digestibility. Besides, supplementation with PFB presented a linear reduction in *Escherichia coli* (*p* = 0.003), accompanied by linear improvement in *Lactobacillus* spp. (*p* < 0.001) and serum high density lipoprotein‐cholesterol (HDL‐C; *p* = 0.001). Broken‐line analysis indicated optimal PFB supplementation levels of 0.60, 0.47, and 0.60 g/kg diet for *E. coli, Lactobacillus* spp., and HDL‐C, respectively. However, feed intake, serum triglycerides, other cholesterols, total protein, albumin, globulin, and uric acid remained unchanged across the groups. Furthermore, supplementation with PFB with an estimated breakpoint of 0.55 g PFB/kg of diet effectively (*p* ≤ 0.02) reduced serum liver enzymes (aspartate aminotransferase, alanine aminotransferase, and alkaline phosphatase) indicating better liver health. In addition, PFB supplementation linearly reduced meat ether extract content and improved meat redness (*p* < 0.001), with optimal responses at 0.58 and 0.60 g/kg diet, respectively, while having no impacts on meat protein, ash, lightness, or yellowness. Therefore, dietary inclusion of PFB at 0.55 g–0.60 g per kg diet optimized growth performance through enhanced weight gain, feed efficiency, and nutrient digestibility, while favorably modulating cecum microbiome, serum cholesterol, liver enzyme activities, and ameliorated meat quality.

## Graphical abstract

Plant‐based flavonoid blend (PFB) at 0.60 g/kg diet produced the best growth performance, feed efficiency, and dry matter digestibility in growing rabbits. It also improved gut microbiome balance and increased serum high‐density lipoprotein‐cholesterol. Liver health was enhanced through lower enzyme activity at an estimated breakpoint of 0.55 g PFB/kg diet. PFB improved meat redness and reduced crude‐fat content. Overall, PFB at 0.55–0.60 g/kg diet improved performance, gut health, serum beneficial metabolite, liver function, and meat quality without adverse effects on meat traits.

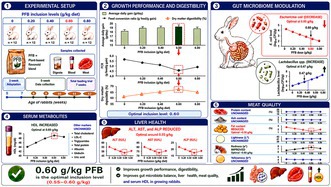

## 1 Introduction

Rabbit meat has good nutritional properties like an abundance of digestible protein, minimal fat content, high unsaturated fatty acid content, low cholesterol, and high vitamin B_12_ and E (Bakeer et al. 2020). It possesses a higher concentration of most minerals except sodium, which is a reason for considering this meat more appealing to people with high blood pressure (Siddiqui et al. 2023). With global concern for healthier and more sustainable diets, the acceptance of rabbit meat is gaining attention, as consumers perceive rabbit meat to be healthier than other meat and fish (Petrescu and Petrescu‐Mag 2018). At the same time, rabbit farming, either on a large or small scale, reflects its economic and beneficial impact on the world market. To produce quality meat for consumers, emphasis is now placed on the use of safe feed additives derived from natural sources like plant‐derived flavonoid (Bakeer et al. 2021).

Flavonoids are plant‐derived polyphenolic compounds widely present in fruits, vegetables, and cereals, known for their antioxidant, antibacterial, and anticancer properties (Adli et al. 2024). In rabbits, dietary sea buckthorn flavonoids (0.10–0.40 g/kg) show dose‐dependent effects, with 0.20 g per kg optimizing growth, feed efficiency, carcass traits, and lipid metabolism, likely via improved antioxidant status and immunity (Du et al. 2023). Similarly, ginkgo leaf extract (4.0 g per kg diet), rich in flavonoids such as quercetin, enhances antioxidant capacity and catalase activity, reducing oxidative stress. This limits myoglobin oxidation and lipid peroxidation, thereby improving meat color and reducing fat deposition (Lu et al. 2025). In addition, feeding a 1.2 mL herb mixture per kg diet, comprising eucalyptus oil (0.2 mL), marjoram oil (0.2 mL), and Artemisia extract (0.8 mL), improved fiber digestibility, live weight, and feed efficiency compared with feeding each herb alone (Dahy et al. 2026).

Plant‐based flavonoids blend (PFB), composed of hops, liquorice, and Arabic gum, is normally used as a growth stimulating supplement in chicken (Akter et al. 2024; Kazi Agro Ltd 2022). Its primary ingredient, hops ( *Humulus lupulus* L.) is rich in flavonoids, mainly quercetin, rutin, luteolin, and xanthohumol (Khaliullina et al. 2024), which possess antimicrobial and anti‐inflammatory properties. Supplementation of hop extract xanthohumol at 10 g per kg diet improves the growth performance and meat quality by suppressing the meat oxidation and meat fat content in rabbits (Lacková et al. 2025). Alike hops, *Artemisia franciscana* extract, rich in flavonoids namely rutin and quercetin, improves antioxidant status and tissue health in female rabbits (Higazy et al. 2026). Besides, supplementation with liquorice extract at 0.10 g per kg diet displays in increased weight gain, improved nutrient digestibility, ameliorated meat quality, more favorable serum lipid constituents, and liver enzymes in rabbits (Alatrony et al. 2022). In contrast, higher inclusion levels (2.0, 4.0, or 6.0 g per kg diet) fail to enhance rabbit growth performance but are associated with improved meat shelf life, as evidenced by in vitro assessments (Dalle Zotte et al. 2020). Supplementation of Arabic gum at 1.0–2.0 g per kg diet (El‐Ratel et al. 2019) or 0.4–1.0 g per liter in drinking water (Aldawood et al. 2025) improves performance, beneficial serum metabolites, antioxidant status, immune responses, and favorable gut microbiota in rabbits under heat stress conditions. However, to date, there is a paucity of published literature investigating the effects of this herbs blend known as PFB in mammalian species particularly in rabbits. In a study, Akter et al. (2024) reveal the beneficial effect of this PFB at 0.60 g per kg diet on broiler performance, nutritional digestibility, blood biomarkers, and meat quality. However, there is a lack of findings on the consequence of pure PFB on the performance, meat quality, and specific dose requirements for pure PFB in growing rabbits. Based on these circumstances, current research was conducted to investigate how PFB supplementation at different doses affects the performance, nutrient digestibility, cecum microbial status, blood biomarkers, liver enzymes, and meat quality of growing New Zealand White rabbits.

## 2 Materials and Methods

### 2.1 Ethics Statement

The experimental protocol for rabbit handling and samples collection was approved by the Ethical Standards of the Research Committee (ESRC) at the Bangladesh Agricultural University Research System (BAURES), BAU, Mymensingh, Bangladesh (BAURES/ESRC/AH/71).

### 2.2 Rabbits, Experimental Design, Diet, and Management

The experiment was conducted at the Rabbit Unit in the field laboratory under the Animal Nutrition Department, Faculty of Animal Husbandry, Bangladesh Agricultural University, Mymensingh, Bangladesh. A total of 60 New Zealand White weaned rabbits, 36 days old, with an average body weight (BW) of 552.63 ± 13.44 g, were randomly assigned to five dietary groups. Each group was assigned to 12 replicates, with one rabbit per cage, following a completely randomized design for a duration of 7 weeks. After 2 weeks of diet adjustment period, the data collection period continued for the next 5 weeks to end at 12 weeks of age. The experimental feeding period aligns with previous study where growth performance and meat quality in rabbits have been evaluated over a 6 weeks feeding period (Birolo et al. 2020). Furthermore, under typical Bangladeshi production and marketing conditions, rabbits are commonly slaughtered at a live body weight of approximately 1.20–1.40 kg (Uddin et al. 2014; Chowdhury et al. 2022).

The basal diet was formulated as a total mixed ration (TMR) and was designed to meet the nutritional requirements of rabbits as specified by De Blas and Wisewan (2020). The plant‐based flavonoid blend (PFB), namely Anta Phyt Optidose (Dr. Eckel Animal Nutrition GmbH and Co. KG Im Stiefelfeld 10, Niederzissen, Germany), was procured via Kazi Agro Ltd., Bangladesh. This PFB is a guaranteed commercial flavonoid preparation derived exclusively from plant extract sources, namely hops ( *Humulus lupulus* L.), licorice ( *Glycyrrhiza glabra* ), and Arabic gum ( *Acacia senegal* ). It was incorporated at inclusion levels of 0.0 (control), 0.20, 0.40, 0.60, and 0.80 g per kg of the TMR basal diet. To prepare the TMR, all concentrate ingredients (Table 1) were procured from a local feed market, while Dhal grass ( *Hymenachne amplexicaulis* ) was cultivated using standard agronomic practices and harvested at 45 days. The grass was then sun‐dried at 30°C–35°C for 1–3 days. Grass, mustard oil cake, and maize seed were ground using a locally manufactured grinder with a screen of 1 mm. Feed items, including roughage and concentrates, were mixed well at 1450 rotations per minute for 15 min in a paddle‐style horizontal mixer manufactured locally, adding 10% water while mixing. Pellets of TMR were subsequently produced by a semiautomated pellet machine having a 4 mm die and sun‐dried until properly bonded.

**TABLE 1:** Ingredients proportion and chemical composition of total mixed ration (TMR).

Ingredients	Basal diet (% on DM)
Dhal grass^a^	32.15
Maize crushed	24.00
Wheat bran	19.60
Soybean meal	17.00
Mustard oil cake	4.90
Molasses	1.50
DL‐methionine	0.10
Mineral‐vitamin premix^b^	0.25
Sodium chloride	0.50

Chemical composition (% on DM)	Basal diet	PFB
DM	87.31	95.70
Crude protein	17.02	3.70
Crude fiber	14.64	6.38
Ether extract	3.15	3.10
Ash	7.43	69.29
Metabolizable energy^c^ (MJ/kg DM)	11.60	—
TPC (mg GAE/g PFB)	—	12.88
TFC (mg QE/g PFB)	—	27.91
TAC (mg AAE/g PFB)	—	6.11

Abbreviations: %, percentage; —, not presented; AAE, ascorbic acid equivalent; DM, dry matter; g, gram; GAE, gallic acid equivalent; kg, kilogram; PFB, plant‐based flavonoid blend; QE, quercetin equivalent; TAC, total antioxidant capacity; TFC, total flavonoid content; TPC, total phenolic content.

^a^ Crude protein, 11.09%; crude fiber, 31.97%; ether extract, 3.38%; ash, 12.04%.

^b^ Each kg vitamin‐mineral premix contained: vitamin A palmitate, 6600 IU; cholecalciferol, 2200 IU; menadione dimethylpyridine bisulfite, 2.2 mg; riboflavin, 4.4 mg; pantothenic acid, 13 mg; niacin, 40 mg; choline chloride, 500 mg; biotin, 1 mg; vitamin B12, 22 μg; ethoxyquin, 125 mg; iron, 50 mg; copper, 6 mg; zinc, 40 mg; manganese, 60 mg; selenium, 0.2 mg.

^c^ Calculated value.

Rabbits were kept individually in steel cages (length 40 cm, width 35 cm, and height 40 cm) containing metal feeders along with bottle drinkers. Standard management and a hygienic environment were maintained throughout the experimental period to minimize stress. TMR and fresh water were supplied *ad libitum* during the whole study period. The PFB was incorporated into the basal diet during the preparation of the TMR.

### 2.3 Data and Samples Collection

The data collection was conducted for a period of 5 weeks. Body weight (BW) and feed intake were noted weekly and daily, respectively. The deviation between the final BW and the initial BW was used to compute BW gain. Average daily gain (ADG) was determined by dividing BW gain by the total number of experimental days (i.e., 35 days). Feed conversion ratio (FCR) was calculated as the proportion of ADG to the average daily feed (fresh TMR) intake. Additionally, growth velocity (GV) was determined by subtracting initial BW from final BW and dividing the result by initial BW (Chowdhury et al. 2022). The formulae for the BW gain, ADG, FCR, and GV are given below:

BWgain=FinalBW–InitialBW

$$\mathrm{ADG}=\mathrm{BW}\;\text{gain}÷\text{Total number of experimental days}\;\left(35\;\text{days}\right)$$

$$\mathrm{FCR}=\text{Fresh}\;\mathrm{TMR}\;\text{intake}\;\left(g\right)/\mathrm{BW}\;\text{gain}\;\left(g\right)$$

GV=BWgain÷InitialBW

All rabbits were reared under humane conditions, receiving proper care, and undergoing regular health monitoring throughout the trial period. At the end of the trial, all rabbits were weighed, and five rabbits per group (total, *n* = 25) were randomly selected for humane sacrifice. Subsequently, blood, meat, and cecal digesta samples were collected following the procedures described by Chowdhury et al. (2022) and Rahman et al. (2022). Immediately after blood collection, it underwent centrifugation at 3410 × g for 15 min for serum separation, which was stored in Eppendorf tubes at −20°C for further analysis (Rahman et al. 2024). The muscle known as *Longissimus thoracis et lumborum* (LTL) was collected for meat color and proximate composition determination. After proper cleaning with water and gentle rubbing using a soft cloth, the LTL muscle samples were stored at 4°C for 24 h to determine meat pH and color. In addition, separate LTL muscle samples were stored around −20°C for subsequent proximate composition analysis. Feces and cecum digesta samples were collected separately from each rabbit to determine the apparent nutrient digestibility and cecum microbiome count, respectively.

### 2.4 Apparent Digestible Nutrient Determination and Samples Analysis

The digestibility trial was conducted over the final 7 days of the trial, using five randomly selected rabbits (total, *n* = 25) from each dietary group. During this time, daily pellet intake was recorded. Fecal samples were collected, weighed, thoroughly mixed, and stored at −4°C. Additionally, a 10 g fecal sample was collected daily to determine fecal dry matter (DM) content. After the 7 days collection period, the fecal sample was homogenized, dried at 60°C in an oven, and ground for further analysis. The dried fecal samples were then sieved through a 1 mm mesh screen and kept at −4°C until the next evaluation (Rahman et al. 2024). Following the standard procedure (Association of Official Analytical Chemists (AOAC) 2005), experimental diets, PFB, feces, and meat (LTL muscle) samples were examined for the determination of DM, ash, crude protein (CP), and ether extract (EE). In detail, DM content of these samples was determined using a micro‐oven at 105°C for 24 h, while ash content was assessed using a muffle furnace at 550°C for 5 h. The CP content of all samples was determined using the Kjeldahl technique, whereas the Soxhlet device was used to determine the EE % in all samples. In addition, crude fiber (CF) contents in diets, feces, and PFB were measured by digesting samples in 1.25% H_2_SO_4_ and then 1.25% NaOH (Association of Official Analytical Chemists (AOAC) 2005). Then, the obtained value of CP, ash, EE, and CF was converted into percentages in DM. The nitrogen free extract (NFE) was subsequently calculated by difference on a DM basis using the sum of CP, EE, CF, and ash, in accordance with Rahman et al. (2026). Additionally, experimental diets and PFB were analyzed for total phenolic content (TPC) and total flavonoid content (TFC) by following standard protocols (Al Islam et al. 2025; Rahman et al. 2021). In brief, approximately 80 mg of TMR diets or PFB samples were extracted separately with 10 mL of 80% methanol and incubated at ambient temperature for 24 h. The extracts were then centrifuged at 6000 × g for 10 min using a centrifuge (Z 306, Hermle, Germany). Then, 0.6 mL of the supernatant was transferred into a 10 mL volumetric flask and mixed with 3 mL of Folin–Ciocalteu reagent, followed by shaking for 5 min for the determination of TPC (expressed as gallic acid equivalent). Afterwards, 2.4 mL of 7.50% sodium carbonate was added to the prepared solution, followed by incubation under dark conditions for 30 min. In addition, pure gallic acid was used to prepare a series of standard solutions for constructing the calibration curve. Finally, the absorbance of the samples and gallic acid standards was measured at 765 nm using an UV visible spectrophotometer (T60; PG Instruments, UK). The colorimetric aluminum chloride protocol, based on flavonoid‐aluminum complex formation, was used to determine the TFC (expressed as quercetin equivalent). The extracted sample (300 μL) was mixed with 10% aluminum chloride (300 μL), 1 M potassium acetate (300 μL), and distilled water (825 μL). The resulting mixture was incubated at room temperature for 40 min. Quercetin solutions of varying concentrations were prepared as standards to generate a calibration curve. Subsequently, the absorbance of both the samples and standard solutions was measured at 415 nm using a similar T60 spectrophotometer. Total antioxidant capacity (TAC) of the diets and PFB samples was determined and expressed as ascorbic acid equivalent with slight modifications according to Al Islam et al. (2025). Briefly, standard solutions of ascorbic acid at varying concentrations were prepared to construct a calibration curve. An aliquot of the sample extract (0.1 mL) was mixed with 1.0 mL of reaction reagent (0.6 M sulfuric acid, 28 mM sodium phosphate, and 4 mM ammonium molybdate). The mixture was incubated in a water bath at 95°C for 1 h and subsequently cooled to ambient temperature. For the blank, 0.1 mL ethanol was used in place of the sample extract. The absorbance of the reaction mixture was then measured at 695 nm using a similar spectrophotometer followed by shaking (Baroi et al. 2024).

On slaughtering day, fresh cecal digesta samples were aseptically collected from each rabbit and processed for bacterial enumeration in accordance with Akter et al. (2024). Briefly, 1 g of digesta sample was diluted by means of the 10‐fold serial dilution way with sterile phosphate buffer solution, and the 10^−5^ dilution was used for drop plating on different agar media. These agar media (HiMedia, Mumbai, India) namely, plate count (Ref no. M091‐500G), eosin methylene blue (Ref no. M317‐500G), and De Man Rogosa and Sharpe (Ref no. M641‐500G) were used to count total viable count (TVC), *Escherichia coli* , and *Lactobacillus* spp., respectively. The usual aerobic incubation period was 24 h at 37°C, but a 48 h incubation period was applied for *Lactobacillus* spp. in the presence of limiting level of oxygen. The bacterial numbers were manually counted and reported as log_10_ colony‐forming units per g of fecal.

At first, serum samples were thawed, and reagents were equilibrated to room temperature prior to use. A bio analyzer (Urit‐810, Chemistry Analyzer, Urit Medical Electronic Co. Ltd., Gullin, Guangxi, 541001, China) was used to examine the serum lipid profiles, protein indices, and liver enzymes' activity. According to the supplied standard protocol, each kit was used to calculate the correction factor. In detail, 1.0 mL of triglycerides (TG) or total cholesterol (TC) reagent (Human Company, USA) was taken in separate cuvettes, then mixed with 10 μL serum and incubated at room temperature for 10 min. The absorbance for TG and total cholesterol was measured separately at 546 nm against a reagent blank at 37°C. For high density lipoprotein‐cholesterol (HDL‐C) determination, 1.0 mL of reagent (Human Company, USA) was mixed with 0.50 mL of serum and centrifuged (Hermle Z 306, Germany) at 5000 × g for 10 min. The resulting supernatant (50 μL) was transferred to 1.0 mL of reagent, incubated at room temperature for 10 min, and absorbance was measured at 546 nm at 37°C. The low density lipoprotein‐cholesterol (LDL‐C) and very low density lipoprotein‐cholesterol (VLDL‐C) were determined by following the equation mentioned by Rahman et al. (2024). Using the same manufacturer's kits and their protocol, 20 μL of serum was mixed with 1.0 mL of either Biuret reagent (total protein) or uric acid reagent (uric acid) and incubated at room temperature for 10 min. The absorbance was measured at 546 nm against a reagent blank at 37°C. For the determination of serum albumin, 10 μL of serum was mixed with 1.0 mL of reagent. Then the resulting mixture was incubated at room temperature for 5 min and absorbance was measured at 546 nm against a reagent blank at 37°C. Serum globulin was calculated by subtracting albumin from total protein (Rahman et al. 2024). The concentrations of aspartate aminotransferase (AST) and alanine aminotransferase (ALT) were measured using spectrophotometric techniques in accordance with the manufacturer's instructions using commercial enzymatic assay kits from Sigma Aldrich (Kat no. C9033, Sigma Aldrich, Germany) and Biovision (Kat no. K753, BioVision, South Milpitas Boulevard, Milpitas, CA 95035, USA), respectively. Serum alkaline phosphatase (ALP) concentration was measured by incubating 20 μL of serum with 1.0 mL of ALP reagent (Linear, Spain) at room temperature for 1 min, followed by Urit‐810 bioanalyzer reading at 405 nm against a reagent blank at 37°C.

The LTL meat samples were removed from 4°C storage and allowed to equilibrate to room temperature before analysis. Then, the pH of LTL meat was measured at the first lumbar vertebra level at 24 h using a pH meter (Hanna Instruments, HI 9126, Woonsocket, RI, USA). Standard pH 4 and 7 buffers were used to calibrate the pH meter, and the calibration's consistency was observed between samples. Meat color was determined by colorimeter (Minolta CR‐410 colorimeter, Minolta Camera Co., Osaka, Japan) after 24 h. For meat color, a single measurement consists of three subsequent flashes of illumination to get the average value. The L*, a*, and b* values were noted to get the values of lightness, redness, and yellowness. Then, the saturation index (SI) was calculated by adopting a formula, SI = √(*a* ^2^ + *b* ^2^) according to Rahman et al. (2022).

### 2.5 Statistical Analysis

Microsoft Excel was applied to organize the raw data, and IBM SPSS (version 22.0, IBM Crop., USA) was used for statistical analysis. A one‐way analysis of variance (ANOVA) was conducted using the following model:

Yij=μ+fi+eij

Here, Yij = response of the rabbits (response variables such as ADG, FCR, …), μ = overall mean, fi = fixed effect of PFB supplementation (dietary groups), and eij = residual error.

To compare means among dietary groups, Tukey's multiple comparison test was applied, and differences at *p* < 0.05 were considered statistically significant. In addition, orthogonal polynomial contrasts were used to test linear, quadratic, and cubic trends in the responses across increasing dietary PFB doses.

Then, to quantify the dose‐dependent effects of PFB, a one‐breakpoint broken‐line (segmented regression) model was fitted in R (version 4.5.1). This following model was specified as:

$$Y=\alpha +\beta_{1}X+\varepsilon ,\;X\leq \phi$$

$$Y=\alpha +\beta_{1}X+\beta_{2}\left(X-\phi \right)+\varepsilon ,X>\phi$$

where, Y = response variables, X = dietary PFB doses, α = intercept, β_1_ = slope before the breakpoint, β_2_ = change in slope after the breakpoint, *ε* = error term, and ϕ = estimated breakpoint (optimal PFB dose). Then, the optimal inclusion (minimum to maximum) range of PFB was estimated in R (version 4.5.1) from the broken‐line breakpoint and its standard error, using the 95% confidence interval.

## 3 Results

### 3.1 Growth Performance

Dietary supplementation of PFB had no effect on initial BW of rabbits (*p* = 0.99), indicating similar baseline conditions among dietary groups (Table 2). However, growth performance variables were significantly influenced by dietary groups. Final BW increased significantly with flavonoid supplementation (*p* = 0.01), with rabbits receiving 0.60 g PFB per kg diet showing the optimum value, accompanied by significant linear (*p* = 0.003), quadratic (*p* = 0.04), and cubic (*p* = 0.03) responses. Similarly, BW gain and ADG were significantly improved (*p* < 0.001), with the greatest responses observed at 0.60 g PFB per kg diet, showing significant linear, quadratic, and cubic trends.

**TABLE 2:** Influence of different doses of plant‐based flavonoid blend supplementation through TMR on performance of growing rabbits.

Variables	Plant‐based flavonoid blend (g/kg diet)					SEM	*p*			
	0.00	0.20	0.40	0.60	0.80		OWA	L	Q	C
Initial BW (g/R)	554	552	555	553	549	3.47	0.99	0.74	0.79	0.79
Final BW (g/R)	1205^b^	1219^b^	1231^ab^	1267^a^	1233^ab^	6.40	0.01	0.003	0.04	0.03
BW gain (g/R)	651^d^	667^c^	676^bc^	714^a^	684^b^	5.66	< 0.001	< 0.001	< 0.001	0.001
ADG (g/R)	18.60^d^	19.06^c^	19.31^bc^	20.40^a^	19.55^b^	0.16	< 0.001	< 0.001	< 0.001	0.001
Growth velocity	1.18^b^	1.21^ab^	1.22^ab^	1.29^a^	1.25^ab^	0.01	0.04	0.01	0.29	0.21
DFI (TMR; g/R)	96.98	97.41	96.00	97.04	95.49	0.34	0.38	0.19	0.60	0.76
DDMI (TMR; g/R)	84.67	85.05	83.82	84.72	83.37	0.30	0.38	0.19	0.60	0.76
FCR (DFI/ADG)	5.21^a^	5.11^ab^	4.97^bc^	4.76^d^	4.88^cd^	0.05	< 0.001	< 0.001	0.03	0.01

*Note:* Superscript letters ^a–d^ means values with dissimilar superscripts differ significantly (*p* < 0.05); Values are means of 12 rabbits per group.

Abbreviations: ADG, average daily gain; BW, body weight; C, cubic; DDMI, daily dry matter intake; DFI, daily fresh intake; g, gram; kg, kilogram; L, linear; OWA, one way ANOVA; Q, quadratic; R, rabbit; SEM, standard error of the means; TMR, total mixed ration.

Growth velocity was also enhanced by flavonoid supplementation (*p* = 0.04), with the optimum GV recorded at 0.60 g PFB per kg diet and a significant linear response (*p* = 0.01). In contrast, daily fresh intake (DFI) and daily dry matter intake (DDMI) were not affected (*p* = 0.38), indicating that dietary groups did not alter feed consumption. FCR was significantly better (*p* < 0.001), with rabbits fed 0.60 g PFB per kg diet exhibiting the lowest FCR values. Significant linear (*p* < 0.001), quadratic (*p* = 0.03), and cubic (*p* = 0.01) responses were observed.

According to the Broken‐line analysis (Table 6), final BW, BW gain, ADG, GV, and FCR all exhibited estimated break‐points at 0.60 g per kg diet, with relatively high coefficients of determination (*R* ^2^ = 0.55–0.89). Among these variables, BW gain and ADG showed the highest model fit (*R* ^2^ = 0.89).

### 3.2 Apparent Nutrient Digestibility and Cecum Microbial Status

Dietary supplementation of PFB significantly influenced apparent nutrient digestibility in growing rabbits (Table 3). DM digestibility increased significantly (*p* < 0.001), exhibiting linear (*p* < 0.001), quadratic (*p* = 0.001), and cubic (*p* = 0.02) responses, with the optimum digestibility observed at 0.60 g PFB per kg diet. CP digestibility was not affected (*p* = 0.27), whereas CF digestibility significantly improved (*p* = 0.01) with a positive linear response (*p* = 0.03). Ether extract (EE) digestibility was also significantly enhanced (*p* = 0.02), showing a quadratic response (*p* = 0.004), while NFE digestibility increased significantly (*p* < 0.001) with linear, quadratic, and cubic trends (*p* ≤ 0.05). The optimum digestibility value for CF, EE, and NFE was generally observed at 0.60 g PFB per kg diet.

**TABLE 3:** Influence of different doses of plant‐based flavonoid blend supplementation through TMR on apparent nutrient digestibility and cecum microbial status in growing rabbits.

Variables	Plant‐based flavonoid blend (g/kg diet)					SEM	*p*			
	0.00	0.20	0.40	0.60	0.80		OWA	L	Q	C
Apparent nutrient digestibility (%)										
Dry matter	60.16^c^	60.99^bc^	61.42^bc^	64.39^a^	61.58^b^	0.40	< 0.001	< 0.001	0.001	0.02
Crude protein	63.47	63.32	64.64	65.67	64.05	0.37	0.27	0.19	0.29	0.13
Crude fiber	30.47^b^	31.16^b^	30.38^b^	32.40^a^	31.01^ab^	0.22	0.01	0.03	0.25	0.07
Ether extract	75.76^b^	78.61^ab^	81.09^ab^	81.92^a^	77.57^ab^	0.75	0.02	0.09	0.004	0.22
NFE	73.52^b^	74.29^b^	73.43^b^	84.43^a^	77.74^b^	1.20	< 0.001	0.001	< 0.001	0.05
Microbiota status (Log_10_ CFU/g cecum digesta)										
pH	6.31	6.21	6.30	6.17	6.14	0.06	0.42	0.20	0.78	0.48
Total viable count	7.75^a^	6.32^b^	6.55^b^	6.57^b^	6.11^b^	0.20	0.04	0.02	0.19	0.07
*Escherichia coli*	4.68^a^	4.21^ab^	3.11^b^	2.22^cd^	1.92^d^	0.39	0.04	0.003	0.82	0.55
*Lactobacillus* spp.	4.20^c^	5.21^bc^	6.94^ab^	7.22^a^	7.13^a^	0.36	0.001	< 0.001	0.04	0.43

*Note:* Superscript letters ^a–d^ means values with dissimilar superscripts differ significantly (*p* < 0.05); Values are means of 5 rabbits per group.

Abbreviations: %, percentage; C, cubic; CFU, colony forming unit; L, linear; NFE, nitrogen free extract; OWA, one way ANOVA; Q, quadratic; SEM, standard error of the means; TMR, total mixed ration.

Plant‐based flavonoid blend (PFB) supplementation also significantly modulated cecum microbial populations (Table 3). Cecum pH remained unchanged (*p* = 0.42), whereas TVC decreased significantly (*p* = 0.04) with a linear response (*p* = 0.02). *E. coli* populations were significantly reduced (*p* = 0.04), showing a linear decrease (*p* = 0.003), with the lowest count observed at 0.80 g per kg diet. In contrast, *Lactobacillus* spp. populations increased significantly (*p* = 0.001), displaying both linear (*p* < 0.001) and quadratic (*p* = 0.04) responses, with the highest values observed at 0.60 and 0.80 g PFB per kg diet.

Broken‐line analysis result (Table 6) indicated that estimated break‐points for nutrient digestibility ranged from 0.55 g to 0.60 g per kg diet. Digestibility of DM, CF, and NFE displayed optimum responses at 0.60 g PFB per kg diet, while EE digestibility was at 0.55 g PFB per kg diet. Dry matter digestibility exhibited the strongest predictive relationship (*R* ^2^ = 0.77). For gut microbial parameters, the estimated break‐point for *E. coli* reduction was at 0.60 g PFB per kg diet, while *Lactobacillus* spp. reached its optimum response at 0.47 g PFB per kg diet. The *Lactobacillus* spp. model demonstrated a relatively strong predictive capacity (*R* ^2^ = 0.80).

### 3.3 Blood Biomarkers and Liver Enzymes

Dietary supplementation of PFB exerted selective effects on blood biochemical parameters of growing rabbits (Table 4). Among lipid profile variables, TG, TC, LDL‐C, and VLDL‐C were not significantly affected by dietary groups (*p* > 0.05), although linear responses were observed for TG (*p* = 0.05) and TC (*p* = 0.01). In contrast, HDL‐C increased significantly (*p* = 0.002) with a linear response (*p* = 0.001), reaching the highest value in rabbits supplemented with 0.80 g PFB per kg diet. However, HDL‐C was statistically similar between rabbits fed PFB at 0.60 or 0.80 g per kg diet. Total protein, albumin, globulin, and uric acid remained unaffected by dietary groups (*p* > 0.05).

**TABLE 4:** Influence of different doses of plant‐based flavonoid blend supplementation through TMR on blood biomarkers and liver enzymes of growing rabbits.

Variables	Plant‐based flavonoid blend (g/kg diet)					SEM	*p*			
	0.00	0.20	0.40	0.60	0.80		OWA	L	Q	C
Lipid profile (mg/dL)										
Triglycerides	74.34	74.55	74.65	74.70	78.48	0.60	0.12	0.05	0.12	0.31
TC	82.56	83.37	84.57	86.69	88.18	0.78	0.11	0.01	0.69	0.83
HDL‐C	42.11^c^	42.88^b^	44.64^ab^	45.66^ab^	47.81^a^	0.61	0.002	0.001	0.48	0.95
LDL‐C	25.58	25.59	25.00	26.09	24.67	0.30	0.68	0.58	0.68	0.42
VLDL‐C	14.87	14.91	14.93	14.94	15.70	0.12	0.12	0.05	0.12	0.31
Protein indices (g/dL)										
TP	7.58	7.87	7.56	7.34	7.61	0.08	0.44	0.44	0.94	0.09
Albumin	3.20	2.68	2.64	2.83	3.38	0.14	0.42	0.62	0.07	0.92
Globulin	4.38	5.19	4.92	4.51	4.23	0.15	0.22	0.33	0.07	0.24
Uric acid	4.06	4.75	4.20	4.37	4.71	0.22	0.87	0.61	0.99	0.44
Liver enzymes activity										
LW (%)	3.66	3.48	3.09	3.34	3.69	0.08	0.06	0.84	0.01	0.49
ALT (IU/L)	57.68^a^	54.16^a^	48.96^b^	47.10^b^	48.28^b^	1.50	0.02	0.01	0.07	0.32
AST (IU/L)	81.23^a^	79.88^ab^	77.95^b^	75.76^b^	76.16^b^	0.77	0.01	0.002	0.44	0.64
ALP (IU/L)	23.17^a^	21.89^ab^	18.90^b^	17.64^b^	18.95^b^	0.59	0.004	0.001	0.02	0.30

*Note:* Superscript letters ^a–c^ means values with dissimilar superscripts differ significantly (*p* < 0.05); Values are means of 5 rabbits per group.

Abbreviations: %, percentage; ALP, alkaline phosphatase; ALT, alanine aminotransferase; AST, aspartate aminotransferase; C, cubic; HDL‐C, high density lipoprotein‐cholesterol; IU/L, international unit per liter; L, linear; LDL‐C, low density lipoprotein‐cholesterol; LW, liver weight; OWA, one way ANOVA; Q, quadratic; SEM, standard error of the means; TC, total cholesterol; TMR, total mixed ration; TP, total protein; VLDL‐C, very low density lipoprotein‐cholesterol.

Liver‐related parameters showed significant responses to PFB supplementation. Relative liver weight (%) tended to differ among groups (*p* = 0.06) and exhibited a quadratic response (*p* = 0.01). Serum ALT, AST, and ALP activities were significantly reduced (*p* ≤ 0.02), showing linear responses (*p* ≤ 0.01), while ALP also exhibited a quadratic effect (*p* = 0.02). The lowest AST and ALP activities were observed at 0.60 g PFB per kg diet, whereas rabbits receiving 0.40–0.80 g PFB per kg diet showed lower ALT values compared with the control group.

According to the result of broken‐line analysis (Table 6), serum HDL‐C reached an optimum response at 0.60 g PFB per kg diet with a strong model fit (*R* ^2^ = 0.78), whereas serum ALT, AST, and ALP activities exhibited optimal responses at 0.55 g PFB per kg diet with a model fit (*R* ^2^ = 0.55–0.76).

### 3.4 Meat Quality

Dietary supplementation of PFB selectively affected meat composition and color characteristics of growing rabbits (Table 5). Moisture, CP, and ash contents were not significantly influenced by dietary groups (*p* > 0.05), although CP showed a significant cubic response (*p* = 0.02). Meat EE content was significantly reduced (*p* = 0.003), exhibiting a linear decrease (*p* < 0.001) with increasing PFB supplementation, with the lowest value observed at 0.80 g PFB per kg diet. However, meat EE content did not differ significantly between rabbits receiving 0.60 and 0.80 g PFB per kg diet. Regarding meat color traits, pH, L*, and b* remained unchanged (*p* > 0.05). However, meat a* significantly increased (*p* = 0.001), showing a linear response (*p* < 0.001), with the highest values recorded at 0.60 and 0.80 g PFB per kg diet. Similarly, meat SI increased significantly (*p* = 0.003) with a positive linear response (*p* < 0.001), reaching the optimum value at 0.60 g PFB per kg diet.

**TABLE 5:** Influence of different doses of plant‐based flavonoid blend supplementation through TMR on meat quality of growing rabbits.

Variables	Plant‐based flavonoid blend (g/kg diet)					SEM	*p*			
	0.00	0.20	0.40	0.60	0.80		OWA	L	Q	C
Meat composition (fresh basis, %)										
Moisture	74.95	76.52	76.23	76.07	76.69	0.25	0.20	0.08	0.37	0.13
Crude protein	20.72	19.08	19.82	20.92	19.93	0.27	0.17	0.88	0.41	0.02
Ash	1.47	1.37	1.34	1.36	1.40	0.03	0.66	0.48	0.20	0.81
Ether extract	1.86^a^	1.69^ab^	1.50^a^	1.33^bc^	1.21^c^	0.07	0.003	< 0.001	0.73	0.81
Meat color										
pH	6.29	6.12	6.19	6.09	5.98	0.04	0.42	0.20	0.78	0.48
Lightness (L*)	42.22	40.37	42.45	39.95	41.76	0.42	0.23	0.64	0.42	0.89
Redness (a*)	5.95^b^	6.34^b^	6.71^b^	7.63^a^	7.56^a^	0.20	0.001	< 0.001	0.67	0.21
Yellowness (b*)	4.41	4.46	4.45	4.89	4.57	0.09	0.49	0.31	0.85	0.22
Saturation index	7.42^c^	7.76^b^	8.05^b^	9.07^a^	8.83^a^	0.19	0.003	< 0.001	0.63	0.14

*Note:* Superscript letters ^a–c^ means values with dissimilar superscripts differ significantly (*p* < 0.05); Values are means of 5 rabbits per group.

Abbreviations: %, percentage; C, cubic; L, linear; OWA, one way ANOVA; Q, quadratic; SEM, standard error of the means; TMR, total mixed ration.

Broken‐line analysis result (Table 6) revealed that meat EE content reached its optimum reduction at 0.58 g PFB per kg diet (*R* ^2^ = 0.78), while a* and SI showed optimal responses at 0.60 g PFB per kg diet, good predictive relationships (*R* ^2^ = 0.72–0.77). The response patterns suggest that PFB supplementation improved meat quality characteristics up to an inclusion level circa 0.60 g per kg diet.

**TABLE 6:** Determination of optimum break‐point of plant‐based flavonoid blend dose for the optimal growth performance, apparent nutrient digestibility, cecum microbiota, blood biomarkers, liver enzymes, and meat quality in rabbits.

	Plant‐based flavonoid blend (g/kg diet)				Broken‐line regression model			
	Break point	SE	Minimum	Maximum	*R* ^2^	Intercept	Slope	
							Before	After
Performances								
Final body weight	0.60	0.07	0.45	0.75	0.69	1201	99	−137
Body weight gain	0.60	0.04	0.50	0.70	0.89	647	99	−112
Average daily gain	0.60	0.04	0.50	0.70	0.89	18.5	2.83	−3.21
Growth velocity	0.60	0.12	0.33	0.87	0.55	1.17	0.18	−0.16
Feed conversion ratio	0.60	0.06	0.46	0.74	0.87	5.24	−0.75	0.47
Apparent nutrient digestibility								
Dry matter	0.60	0.05	0.49	0.71	0.77	59.77	6.57	−10.63
Crude fiber	0.60	0.12	0.33	0.87	0.36	30.34	2.51	−4.19
Ether extract	0.55	0.06	0.41	0.68	0.65	75.83	13.32	−21.77
Nitrogen free‐extract	0.60	0.12	0.35	0.85	0.52	71.63	15.94	−17.27
Microbiome status								
*Escherichia coli*	0.60	0.12	0.40	0.80	0.59	4.83	−4.24	−1.82
*Lactobacillus* spp.	0.47	0.12	0.21	0.72	0.80	4.08	6.85	−0.45
Blood biomarkers and liver enzymes								
High density lipoprotein‐cholesterol	0.60	0.12	0.33	0.87	0.78	41.96	6.20	10.63
Alanine aminotransferase	0.55	0.09	0.35	0.75	0.61	57.68	−49.4	6.27
Aspartate aminotransferase	0.55	0.09	0.35	0.75	0.55	81.23	−16.8	6.85
Alkaline phosphatase	0.55	0.08	0.38	0.73	0.76	23.17	−10.68	6.55
Meat quality								
Ether extract	0.58	0.05	0.47	0.69	0.78	1.86	−0.90	−0.60
Redness	0.60	0.09	0.40	0.80	0.77	5.85	2.71	0.43
Saturation index	0.60	0.17	0.23	0.96	0.72	7.29	2.62	−0.16

Abbreviation: SE, standard error.

## 4 Discussion

The present study demonstrated that dietary supplementation of a PFB, rich in flavonoids, phenolics, and antioxidant compounds, improved growth performance, nutrient utilization, gut microbial balance, metabolic health, and meat quality in growing rabbits. Broken‐line regression consistently identified an optimal supplementation level of approximately 0.60 g PFB per kg diet, suggesting a biologically relevant threshold beyond which additional benefits were limited. Although the present study identified approximately 0.60 g PFB per kg diet as the optimal supplementation level, responses to dietary flavonoids are not always consistent across studies. Variations in flavonoid source, chemical structure, bioavailability, dietary composition, animal species, age, and physiological status may influence biological efficacy. Such variability may explain why some studies have reported limited or inconsistent responses to flavonoid supplementation despite similar experimental objectives.

Dietary PFB improved final BW, BW gain, ADG, GV, and FCR without affecting DFI and DDMI, indicating that the growth‐promoting effects were primarily driven by enhanced nutrient utilization rather than increased feed consumption. Similar responses have been reported in rabbits and poultry supplemented with flavonoid‐rich feed additives (Hassan et al. 2018; Rashidi et al. 2019; Zhang and Kim 2020; Al‐Baadani et al. 2022). The improvement in growth performance may be attributed to the antioxidant and anti‐inflammatory properties of flavonoids. By reducing oxidative stress and enhancing endogenous antioxidant defenses, including superoxide dismutase, catalase, glutathione peroxidase, and total antioxidant capacity, flavonoids help preserve intestinal and cellular integrity, thereby improving nutrient partitioning toward growth (Lu et al. 2025). Additionally, supplementing rabbits with flavonoid‐ and polyphenol‐rich Arabic gum at 0.4–1.0 g via drinking water improved immunity and liver health, while reducing pro‐inflammatory cytokines such as IL‐1β and TNF‐α (Aldawood et al. 2025). In the present study, inclusion of PFB containing Arabic gum similarly promotes liver health and may improve immune function by attenuating inflammatory cytokine responses, which can enhance nutrient utilization and, consequently, improve growth performance in rabbits.

This interpretation is supported by the enhanced digestibility of DM, CF, EE, and NFE observed in the present study. Improved digestibility may result from better intestinal morphology, enhanced digestive enzyme activity, improved gut barrier function, and modulation of hindgut fermentation (Lucio‐Ruiz et al. 2024). Similar improvements in nutrient utilization have been reported following flavonoid supplementation in rabbits and poultry (Dabbou et al. 2017; Mohammed et al. 2020; Goliomytis et al. 2014). Similar improvements in nutrient utilization have been reported following flavonoid supplementation in rabbits and poultry (Dabbou et al. 2017; Mohammed et al. 2020; Goliomytis et al. 2014). For instance, flavonoid‐rich chicory leaf extract improved villus height, villus width, and crypt depth in rabbits (Hassan et al. 2026). In the present study, dietary PFB did not affect feed intake, but it improved digestibility and growth performance, which may be attributed to flavonoid‐mediated reductions in intestinal inflammation and improved mucosal integrity, thereby enhancing nutrient absorption and utilization. Although higher inclusion levels maintained comparable final BW and feed efficiency, the slight reduction in weight gain at 0.80 g PFB per kg diet suggests diminishing returns, potentially due to reduced bioavailability, metabolic saturation, or prooxidant effects associated with excessive flavonoid intake (Tumilaar et al. 2024; Li et al. 2025).

The improvements in growth performance observed in the present study appear to be the result of interconnected physiological responses rather than a single direct effect of flavonoid supplementation. The favorable modulation of cecal microbiota, characterized by increased *Lactobacillus* spp. and reduced *E. coli* populations, likely enhanced hindgut fermentation and nutrient availability, contributing to the observed improvements in nutrient digestibility. Improved digestive efficiency may subsequently have increased the availability of metabolizable nutrients for tissue accretion and growth. In parallel, the antioxidant and anti‐inflammatory properties of flavonoids may have reduced metabolic costs associated with oxidative stress and immune activation, thereby allowing a greater proportion of absorbed nutrients to be directed toward productive functions. Collectively, these responses support the existence of a gut–metabolism–growth axis through which PFB supplementation improved overall productive performance in growing rabbits.

A major finding of the present study was the favorable alteration of cecum microbial populations, characterized by increased *Lactobacillus* spp. and reduced *E. coli* and TVC. These findings support previous reports in which rabbits received flavonoid‐rich chicory leaf extract (Hassan et al. 2026) and Arabic gum (Aldawood et al. 2025) as feed additives. Such changes are particularly important because gut microbiota plays a central role in nutrient digestion, immune regulation, and intestinal health. The reduction in *E. coli* may be explained by the ability of flavonoids to disrupt bacterial membranes, inhibit nucleic acid synthesis, and interfere with microbial energy metabolism (Xie et al. 2015). Conversely, the increase in *Lactobacillus* spp. supports evidence that flavonoids and their metabolites can exert prebiotic‐like effects that selectively promote beneficial bacteria (Dhiman et al. 2025). Beneficial microbes promote short‐chain fatty acid production, vitamin synthesis, and competitive exclusion of pathogens, thereby improving nutrient absorption and intestinal function (Fathima et al. 2022; Gao et al. 2025). Furthermore, a healthier microbial environment may stimulate intestinal villus development and epithelial regeneration, increasing digestive and absorptive capacity (Prihambodo et al. 2020). Collectively, these findings support a gut‐mediated mechanism through which PFB enhances productive performance.

Dietary PFB supplementation positively influenced metabolic and hepatic health, as evidenced by increased serum HDL‐C concentrations and reduced ALT, AST, and ALP activities. The selective increase in HDL‐C suggests enhanced reverse cholesterol transport rather than broad alterations in lipid metabolism. Flavonoids have been shown to stimulate apolipoprotein A‐I synthesis, improve HDL‐C functionality, and facilitate cholesterol efflux from peripheral tissues to the liver (Millar et al. 2017; Ayoub 2022). In addition, an increased population of lactic acid bacteria in the rabbit intestine in the study may improve serum HDL‐C concentration by modulating cholesterol metabolism, enhancing bile acid metabolism, and promoting a healthier gut microbial balance (Colombino et al. 2022). The reductions in liver enzyme activities indicate improved hepatic integrity and reduced hepatocellular stress. These hepatoprotective effects are likely mediated through the antioxidant and anti‐inflammatory actions of flavonoids, which scavenge reactive oxygen species, stabilize hepatocyte membranes, and suppress inflammatory pathways (Zhao et al. 2018; Al‐Zharani et al. 2023; Liao et al. 2024). Besides, the lower *E. coli* and higher *Lactobacillus* spp. in the current study indicate an improved gut microbial balance in rabbits, which is typically associated with better gut health and reduced pathogenic pressure. This shift may also coincide with more favorable liver enzyme profiles, suggesting reduced serum ALT, AST, and ALP concentration (Tufarelli et al. 2025). Furthermore, the improved metabolic status may also have contributed to the enhanced growth performance and nutrient utilization observed in flavonoid‐supplemented rabbits.

The improvement in hepatic enzyme profiles may also have broader implications for productive performance. The liver serves as a central organ regulating nutrient metabolism, lipid transport, and energy partitioning. Therefore, improved hepatic integrity may facilitate more efficient utilization of absorbed nutrients, contributing not only to improved metabolic status but also to enhanced growth performance and feed efficiency observed in flavonoid‐supplemented rabbits. This interpretation further supports the integrated nature of the physiological responses induced by PFB supplementation.

The beneficial effects of PFB extended to meat quality, particularly through reduced intramuscular fat deposition and improved color characteristics. The reduction in meat EE content suggests that flavonoids modulated lipid metabolism by suppressing adipogenesis and promoting fatty acid oxidation. Previous studies have shown that flavonoids regulate genes involved in lipid catabolism while inhibiting pathways associated with fat synthesis and adipocyte differentiation (Ouyang et al. 2016; Lu et al. 2025). The concurrent increase in HDL‐C further supports enhanced lipid mobilization and utilization. Dietary PFB also increased meat a* and SI, likely through improved oxidative stability of muscle pigments. The antioxidant properties of flavonoids can reduce lipid and myoglobin oxidation, thereby preserving meat color stability and oxidative quality (Cao et al. 2023). Furthermore, alike flavonoid‐rich Arabic gum, PFB may alleviate systemic inflammation by modulating the gut microbiota and health, enhancing immune function, and suppressing pro‐inflammatory cytokine production (Aldawood et al. 2025). These combined antioxidant and immunomodulatory effects may contribute to improved meat red appearance and color stability. Since meat color strongly influences consumer acceptance, these improvements may enhance the marketability of rabbit meat without altering its fundamental nutritional composition.

The consistency of responses across growth performance, nutrient digestibility, gut microbiota, metabolic health, and meat quality indicates that approximately 0.60 g PFB per kg diet is the optimal supplementation level for growing rabbits. At this inclusion rate, PFB effectively enhanced productive performance and product quality while supporting gut and liver health. These findings highlight the potential of PFB as a sustainable functional feed additive capable of improving rabbit production while reducing reliance on antibiotic growth promoters. The present study did not directly evaluate intestinal morphology, antioxidant enzyme activities, inflammatory biomarkers, microbial metabolites, or the expression of genes involved in nutrient transport, lipid metabolism, and immune regulation. Consequently, the proposed mechanisms are primarily inferred from observed physiological responses and supporting literature. Future investigations integrating transcriptomic, metabolomic, and microbiome‐based approaches would provide a more comprehensive understanding of the molecular pathways through which plant‐derived flavonoids influence rabbit productivity, health, and product quality.

## 5 Conclusions

Dietary supplementation of a plant‐based flavonoid blend (PFB) improved growth performance, nutrient utilization, cecum microbial balance, metabolic health, liver function, and meat quality in growing rabbits without affecting feed intake. Importantly, this study provides novel evidence regarding the dose‐dependent effects of PFB in rabbits and identified an optimal supplementation level of approximately 0.55–0.60 g PFB per kg diet. These findings highlight the potential of PFB as a natural functional feed additive for improving rabbit productivity and product quality. Further studies are warranted to elucidate the underlying molecular mechanisms and validate these effects under diverse production conditions.

## Author Contributions

**Md. Aliar Rahman:** methodology, conceptualization, data curation, formal analysis, writing – original draft, writing – review and editing. **Md. Mehedi Hasan:** methodology, data curation, writing – original draft, writing – review and editing. **Md. Abul Hashem:** methodology, resources, writing – review and editing. **Mahbubul Pratik Siddique:** methodology, resources, writing – review and editing. **Rakhi Chowdhury:** conceptualization, methodology, writing – original draft, writing – review and editing, funding acquisition, supervision, resources.

## Funding

This work was supported by University Grant Commissions of Bangladesh, Life Science 51 (2021‐2022).

## Conflicts of Interest

The authors declare no conflicts of interest.

## Supporting information


**Table S1:** Nutrient composition, total flavonoid, phenolic, antioxidant contents of different pellet diets.

## Data Availability

All relevant data underlying the current findings are within the paper. In addition, the nutrient composition, total flavonoid, phenolic, antioxidant contents of different pellet diets were not presented within the manuscript due to minor and insignificant differences. The data are presented in Table S1.
