# Supplementary material for: Dose Titration of Plant‐Based Flavonoid Blend Supplementation on Performance, Digestibility, Gut Microbiome, Blood Biomarkers, and Meat Quality of Growing Rabbits
Source: Food Sci Nutr. 2026 Jul 28;14(8):e72156. doi: 10.1002/fsn3.72156 (PMC13415973; doi:10.1002/fsn3.72156)
Supplement: Supplementary file 1 — Table S1: Nutrient composition, total flavonoid, phenolic, antioxidant contents of different pellet diets. [file FSN3-14-e72156-s001.docx]

**Table S1**

Nutrient composition, total flavonoid, phenolic, antioxidant contents of different pellet diets

| **Chemical composition**  **(% on DM)** | **Plant-based flavonoid blend (g/kg diet)** | | | | |
| --- | --- | --- | --- | --- | --- |
|  | 1. 0 (control or basal diet) | 0.20 | 0.40 | 0.60 | 0.80 |
| Dry matter | 87.31 | 87.45 | 87.34 | 87.26 | 87.30 |
| Crude protein | 17.02 | 16.93 | 17.08 | 16.97 | 17.03 |
| Crude fiber | 14.64 | 14.60 | 14.63 | 14.67 | 14.65 |
| Ether extract | 3.15 | 3.12 | 3.14 | 3.11 | 3.15 |
| Ash | 7.43 | 7.45 | 7.46 | 7.48 | 7.50 |
| Metabolizable energy#  (MJ/kg DM) | 11.60 | 11.60 | 11.61 | 11.60 | 11.60 |
| TPC (mg GAE/ g pellet) | 12.20 | 12.24 | 12.26 | 12.27 | 12.30 |
| TFC (mg QE/ g pellet) | 9.86 | 9.91 | 9.97 | 10.03 | 10.07 |
| TAC (mg AAE/ g pellet) | 8.15 | 8.19 | 8.17 | 8.19 | 8.20 |

PFB, plant-based flavonoid blend; ^#^Calculated value; TPC, total phenolic content; TFC, total flavonoid content; TAC, total antioxidant capacity; GAE, gallic acid equivalent; QE, quercetin equivalent; AAE, ascorbic acid equivalent.
